# Open questions on the environmental chemistry of radionuclides

**DOI:** 10.1038/s42004-020-00418-6

**Published:** 2020-11-12

**Authors:** Gauthier J.-P. Deblonde, Annie B. Kersting, Mavrik Zavarin

**Affiliations:** Physical and Life Sciences Directorate, Lawrence Livermore National Laboratory, Glenn T. Seaborg Institute, Livermore, CA 94550 USA

**Keywords:** Nuclear chemistry, Environmental chemistry

## Abstract

Understanding the biogeochemistry of radionuclides in the environment is essential for effective isolation of nuclear waste in repositories, management of contaminated sites, ensuring long-term protection of our ecosystems, and limiting impacts on human health. Here the authors discuss the extreme complexity of this multidimensional chemistry problem, highlighting the outstanding open questions for the next generations of environmental radiochemists.

Anthropogenic radionuclides are created during nuclear explosions, irradiation in nuclear reactors, particle or X-ray bombardment of materials, and α, β, γ, and ε decay or even fission (spontaneous or neutron induced) of parent radioisotopes. Natural radionuclides are produced by stellar nucleosynthesis and cosmic-ray spallation processes. However, with the exception of the uranium series (notably ^238/234^U and ^226^Ra), natural radioactive isotopes do not pose a significant health concern or environmental perturbation. Anthropogenic radionuclides are produced under extreme pressures, temperatures, and radiation fields, as well as non-equilibrium physicochemical conditions, and this may subsequently influence the surrounding geology and the radionuclides’ fate in the environment. For example, radionuclides deposited in the immediate aftermath of an underground nuclear detonation undergo temperatures that exceed 1,000,000 K, vaporizing ~70 tons and melting another 700 tons of rock for every kiloton of yield^1^. Fortunately, the vast majority of the radionuclides produced by anthropogenic activities (electricity generation, medicine, research, etc.) are short-lived (half-lives <10 years) and the associated waste can be managed by leveraging the natural fast decay of the isotopes. The main concern shared by past, present, and future generations will be the longer-lived (>10 years) radionuclides (lanthanide fission products, ^3^H, ^90^Sr, ^94^Nb, ^99^Tc, ^137^Cs, ^226^Ra, ^233/234/235/236/238^U, ^237^Np, ^239/240/241/242/244^Pu, ^241/243^Am, ^244/245/246/247^Cm, and so on), which will ultimately decay to harmless isotopes too—but only after hundreds or thousands of years. Thus, their potential to adversely impact human health and our environment must be understood at the millennial timescale.

## Radionuclide contamination: a global, complex, and long-lasting issue

**Fig. 1:**
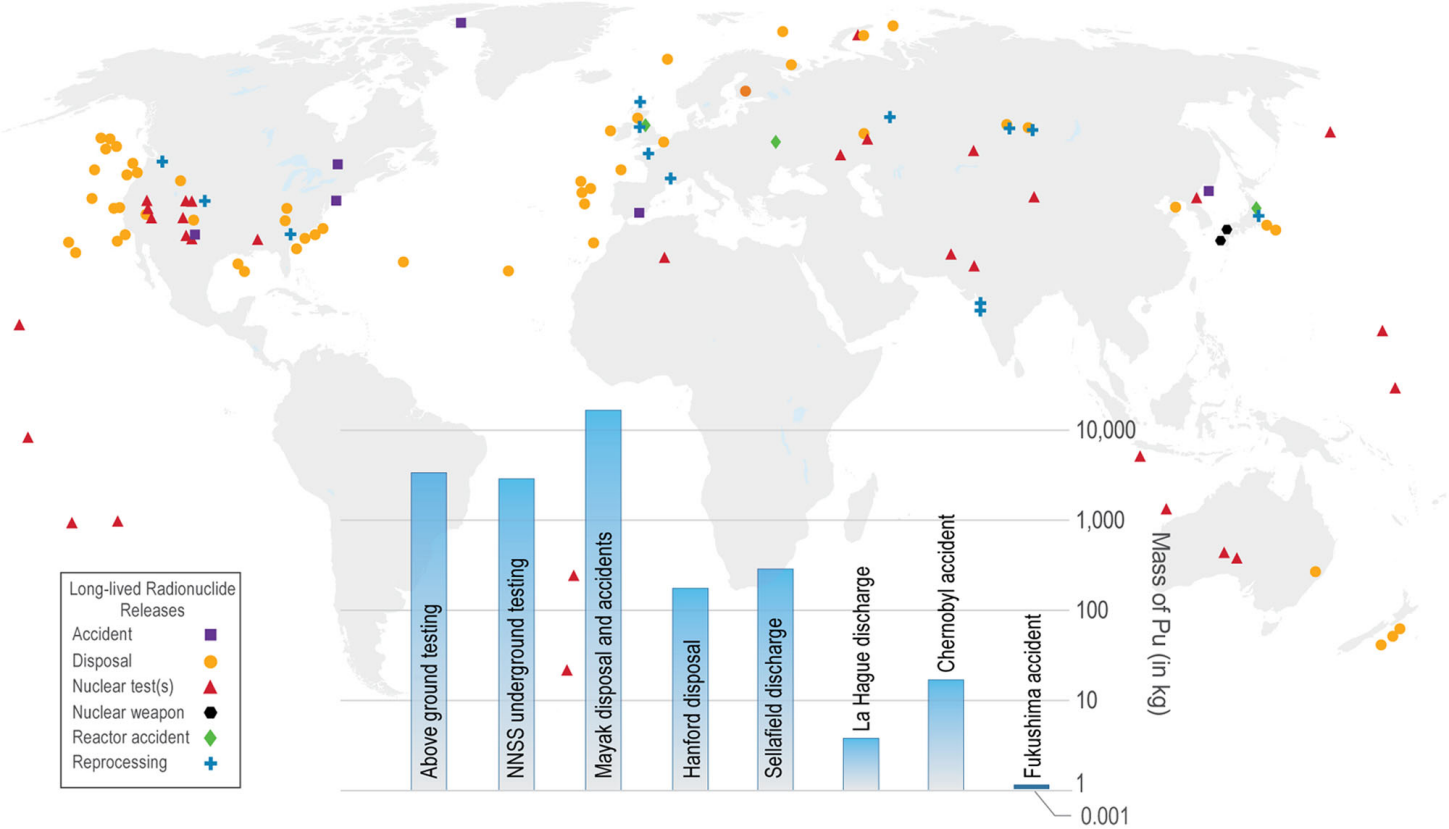
Global view of radiologically impacted sites and Pu inventories at selected sites. Notes: Data taken from Geckeis et al.^2^ and not intended to be comprehensive. Nuclear power generation sites and uranium mining-related sites not included (inset: histogram of Pu inventories at selected sites that are the result of nuclear tests, fuel reprocessing, disposal, discharge, and accidents).
- Radionuclide concentrations of interest span ~15 orders of magnitude, from concentrated fuel rods or solid particles to femtomolar regulatory limits for drinking water standards (e.g., US Environmental Protection Agency Maximum Contaminant Levels for drinking water of 6.4 × 10^–16^, 1.7 × 10^–14^, and 3.7 × 10^–15^ mol/L for ^90^Sr, ^137^Cs, and ^238^Pu, respectively). The variation in concentration and initial form can control the chemical behavior of the radionuclides.
- The behavior of radionuclides involves timescales that are orders of magnitude longer than the typical laboratory experiments (thousands of years versus a few years at best), questioning if short-term experiments (days to weeks) capture the long-term environmental behavior of radionuclides.
- The radionuclides of concern are spread across the periodic table, spanning from light elements that have corresponding stable isotopes in nature (tritium, strontium, etc.) to heavy atoms that are normally not present on earth (technetium, neptunium, plutonium, americium, etc.). Hence, the behavior of nuclear waste is intrinsically difficult to predict due to the wide range of elements they contain, covering almost the entire speciation realm (gas, solids, soluble cations, anions, oxo-species, etc.).
- Environmental systems are inherently complex and the associated radiochemistry may involve solubilized ions, precipitates, aqueous chelators, radicals, mineral phases, colloids, microorganisms, redox changes, and anthropogenic materials (e.g., fuel cladding, waste canister, repository filler, co-contaminants)^3^.

Identifying processes controlling radionuclide migration and predicting their behavior at environmentally relevant timescales (e.g., hundreds or thousands of years) represents one of the most unique challenges that face scientists and society. The problem is inherently interdisciplinary, and involves constraints from geochemistry, biochemistry, mineralogy, inorganic chemistry, material, and nuclear sciences, all of which can be influenced by environmental changes and human intervention. Our understanding of the fate and transport of radionuclides in the environment is limited by the inherent complexity of the problem and challenges associated with radiochemistry, including:

- Long-lived radionuclides (half-lives >10 years) are, by nature, highly hazardous and controlled substances. A restricted availability of some radioisotopes, combined with strict safety protocols for material handling, has limited the number of institutions and scientists willing to embark on environmental radiochemistry research.
- The sites of radionuclide contamination are chemically and geologically diverse, from the atmosphere to the ocean, from surface soils to the deep subsurface, from deserts to the polar regions, resulting in a wide range of biogeochemical conditions influencing their chemical behavior (Fig. 1)^2^.

## Mobility of long-lived radionuclides in the environment: progress and emergent behavior

### Mobility of plutonium

The Comprehensive Nuclear Test Ban Treaty (CTBT) was adopted by the United Nations General Assembly, <25 years ago, on September 10, 1996. While the CTBT had little effect on the accumulation of radioactive waste generated by civilian nuclear activities, it did significantly reduce the direct release of radionuclides into the environment. For example, >6000 kg of Pu was released to the environment during underground and atmospheric nuclear testing^2^. It is estimated that an amount of the same order of magnitude was released globally as a result of poor environmental management of civilian and military activities (Fig. 1). Moreover, an additional ~70,000 kg of Pu are added to the non-released inventory annually for electricity production, yielding a global inventory of 2,630,000 kg Pu as of 2014^2^. In other words, we have taken an almost irreversible path where long-lived radionuclides will exist in our environment for generations to come and must be studied and managed accordingly.

A major breakthrough in the field of environmental radiochemistry was the discovery that sparingly soluble metals can migrate rapidly over several kilometers from their initial source. Kersting et al.^4^ demonstrated—post CTBT—that the migration of radioisotopes, including actinides, over kilometer distances at the Nevada National Security Site (USA) is due to the association between radionuclides and mobile clay and zeolite colloids in suspension in groundwater. Similarly, Novikov et al.^5^ later confirmed long-range (<4 km) colloid-facilitated transport of Pu at Mayak (Russia) due to its association with mobile iron oxide colloids. In a very different scenario, trace concentrations of U and Pu have been detected over a large area (>50 km radial distance) stemming from the Fukushima Daiichi Nuclear Power Plant reactor meltdown accident, 2011 (Japan)^6^. In this case, recent measurements, using advanced microscopy techniques and forensic analysis tools (scanning transmission electron microscopy (TEM), high-resolution TEM, high-angle annular dark-field scanning TEM, scanning TEM energy-dispersive X-ray mapping, secondary ion mass spectrometry, synchrotron micro-focus X-ray fluorescence mapping, and X-ray absorption near-edge spectroscopy) showed that U and Pu are associated with cesium-rich microparticles^5^. These three colloidal/microparticle-facilitated migration mechanisms illustrate the chemical complexity of radionuclide transport.

**Fig. 2:**
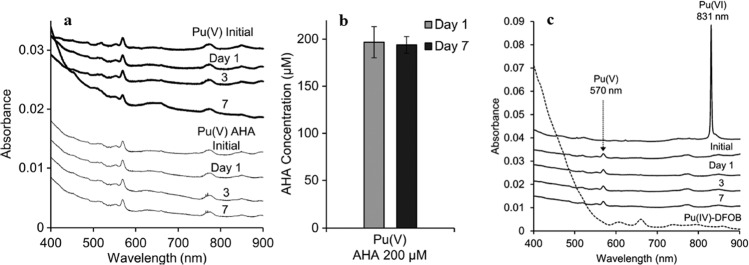
Influence of small organic chelators on Pu redox chemistry. Reaction of 100 μM Pu(V) with 200 μM acetohydroxamic acid (AHA) at pH 5.6. **a** UV–Vis–NIR spectra of Pu(V) control and Pu(V) reacted with AHA showing no reduction to Pu(IV) over 7 days. **b** Concentrations of AHA monitored using GC-MS after 1 and 7 days determined that AHA is not being consumed while reacting with Pu(V). **c** Reaction of 100 μM PuO_2_^2+^ with 200 μM desferrioxamine B (DFOB) at pH 5.5. The UV−Vis–NIR spectra show an immediate reduction of Pu(VI) to Pu(V) that remains stable throughout the experiment. A reference spectra of Pu(IV) DFOB is included showing that the reaction of Pu(VI) with DFOB does not produce any spectral features associated with a Pu(IV) DFOB complex. Adapted with permission from ref. ^7^. Copyright 2018 American Chemical Society.

Very recent progress in the chemistry of actinides and lanthanides also suggest that other phenomena are important in controlling the biogeochemistry of long-lived radionuclides. A recent study by Morrison et al.^7^ demonstrated that some ubiquitous organic chelators, such as water-soluble siderophores, can impact the redox chemistry of radionuclides, strongly influencing their behavior in the environment (Fig. 2). While other recent studies^8,9^ have shown that synthetic multidentate chelators can drive the oxidation state of lanthanides and actinides to +IV, Morrison et al.^7^ demonstrated that even a small bidentate ligand, like acetohydroxamic acid, reduces Pu(VI) to Pu(V) under environmentally relevant conditions, and that the reduction reaction does not produce Pu(IV) or Pu(III). These results raise many questions about the role of commonly occurring natural chelators in environmental samples that are often organic-rich mixtures containing trace amounts of radionuclides.

### Mobility of fission products and early actinides

In the past few years, natural macromolecular systems that are highly selective for the lanthanide (Ln) ions over other salts or co-contaminants have also been identified. Methylotrophic bacteria have been shown^10^ to specifically scavenge and utilize Ln^3+^ ions for essential biological functions. Cotruvo et al.^11^ recently isolated a small protein (Lanmodulin) produced by these bacteria that is even more selective for Ln^3+^ ions than synthetic ligands (stability ratio Ln/Ca, Ln/Zn, Ln/Cu >1,000,000)^12^. Based on these recent developments, the existence of biomacromolecule-mediated transport mechanisms for f-element radionuclides at specific field sites should not be ruled out.

The fundamental chemistry of actinide complexes may also be pressure dependent. Recent results from Sperling et al.^13^ indicate that chemical bonds in some minor actinide complexes become much more covalent at high pressure (1–10 GPa), whereas the equivalent lanthanide species remain almost unaffected. These results suggest that the thermodynamic stability of certain metal contaminants in the subsurface may not conform with behavior observed in the laboratory at atmospheric pressure. Likewise, the influence of elevated subsurface heat is often neglected when modeling the speciation of metal ions in the environment, but two recent studies^14,15^ have demonstrated that phases like ThO_2_, usually considered to be insoluble, or nuclear melt glass produced in nuclear tests can release radionuclides at elevated temperature. Nisbet et al.^14^ found that the solubility of ThO_2_ reaches the parts per million level in hydrothermal solutions at 175–250 °C—orders of magnitude higher that the reference solubility at room temperature. Similarly, Zavarin et al.^15^ demonstrated that nuclear melt glass can release radionuclides at moderately elevated temperatures (~200 °C). Nuclear melt glass was hydrothermally altered for 3 years over a range of temperatures (25–200 °C) and the concentrations of ^60^Co, ^137^Cs, ^152^Eu, and Pu in the fluids were ~100 times higher at the highest temperatures (80–200 °C) compared to those at room temperature. Even after 3 years, very little alteration was observed at the lowest temperatures. These studies highlight the necessity to perform long-term laboratory experiments under temperature and pressure conditions expected at field sites.

### Advances in actinide spectroscopy

Another recent discovery that challenges our understanding of the mobility of radionuclides is the identification of a new metastable solid-phase containing Pu(V), where PuO_2_ was formed from soluble Pu(VI) ions under mildly alkaline conditions. These observations would not have been possible without the use of X-ray absorption near-edge spectroscopy at the M_4_ edge of Pu (compared to the classic but less informative L_3_ edge used for actinide samples). This new spectroscopic technique pioneered by Kvashnina et al.^16^ could help to elucidate new reaction mechanisms for radionuclides, but it also opens the question of the existence of previously unnoticed solid phases.

### Natural long-lived radioisotopes

**Fig. 3:**
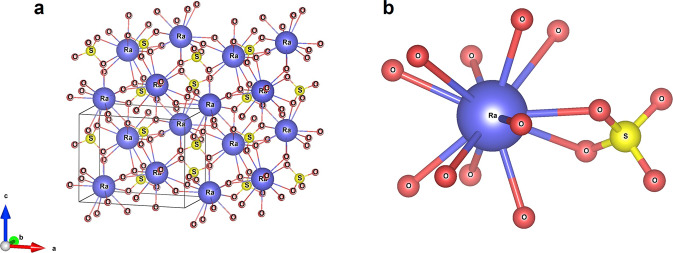
Coordination chemistry of radiotoxic radium. Radium is a natural but highly toxic element for which there is little data available. Its chemistry is peculiar with a high coordination number and weak hydrolysis behavior. Extended crystal structure of ^226^RaSO_4_ (**a**), and local coordination environment of Ra^2+^ in ^226^RaSO_4_ (**b**). Reproduced with permission from ref. ^17^. Copyright 2017 Elsevier.

Naturally occurring radioactive material, particularly the U series isotopes, warrant investigation as they are relatively abundant, some are highly toxic, yet they have received less attention compared to uranium and manmade elements like plutonium. Radium is of particular concern because ^226^Ra is one of the most radiotoxic natural nuclides (specific activity = 37 GBq/g or 1 Ci/g), it decays to radiotoxic radon gas (^222^Rn), and its ion, Ra^2+^, is highly mobile and can replace calcium in bone if ingested. ^228^Ra (*t*_1/2_ = 5.7 years) and ^226^Ra (*t*_1/2_ = 1585 years) are present in the industrial waste from mining and gas operations due to their presence in the ^232^Th and ^238^U decay chains, respectively. Matyskin et al.^17,18^ recently determined the crystal structure of the environmentally relevant radium-barium sulfate, as well as the hydrolysis behavior of Ra^2+^_(aq)_. An exceptionally high coordination number of 12 (compared to 8–10 for other heavy radionuclides, Fig. 3), long bond metal-oxygen distances (2.85–3.35 Å) and weak hydrolysis behavior were observed. These examples underscore the reality that our current understanding of the properties of many radionuclides is limited, but also highlight that new experimental techniques to probe the solid-state and solution-state chemistry of highly radioactive samples offer hope that our limited understanding will soon be a thing of the past.

## Outlook

In the past 20 years, tremendous progress has been made in our understanding of the interactions between radionuclides and the environment. The major open questions that will spark new research in the near future include: the influence of natural organic chelators on the complexation and redox chemistry of metal ions, the role played by macromolecules and microorganisms, the existence of solid phases that control the solubility of radionuclides, and how to perform laboratory experiments that effectively reproduce the behavior of these elements at environmentally relevant (millennial) timescales and physicochemical conditions. Another phenomenon still largely not understood is the formation and/or accumulation of new species (radicals, degradation products, soluble or solid) in the environment induced by the radiation field of nuclear waste (that emit varied radiation: α, β, γ, neutrons, X-rays, etc.) and over a long period of time. Environmental radiochemists will also have to consider the impact of climate change on contaminated field sites, non-nuclear anthropogenic activities, and policy decisions regarding the design of nuclear waste repositories. We hypothesize that these questions can only be answered by stimulating progress in new spectroscopic techniques for radioactive samples, by applying new data science approaches to these complex environmental systems, by encouraging multidisciplinary collaborations, and continuing to motivate the next generation of radiochemists.

In 2021, the Anthropocene Working Group (AWG) plans to submit a proposal to formally adopt a new epoch in geologic time to the International Commission on Stratigraphy, which oversees the official geologic time chart^19^. Although controversial, the AWG is considering ending the Holocene and designating the new “Anthropoene” epoch that coincides with the start of the atomic age, using the world-wide radionuclide signature from the mid-1950s that signals how significantly humans have altered the planet. If adopted, we are, at present, embarking on a new epoch in geologic time that is marked by the presence of anthropogenic long-lived radionuclides. Understanding the impact of anthropogenic radionuclides at geologic timescales will be central to the definition of this new epoch.
